# Theta and beta power in the subthalamic nucleus responds to conflict across subregions and hemispheres

**DOI:** 10.1093/braincomms/fcaf021

**Published:** 2025-01-16

**Authors:** Jessica L Bowersock, Scott A Wylie, Ahmad Alhourani, Ajmal Zemmar, Victoria Holiday, Peter Hedera, Travis Stewart, Elizabeth Bridwell, Isabelle Hattab, Beatrice Ugiliweneza, Joseph S Neimat, Nelleke C van Wouwe

**Affiliations:** Department of Neurological Surgery, University of Louisville, Louisville, KY 40202, USA; Department of Neurological Surgery, University of Louisville, Louisville, KY 40202, USA; Department of Neurological Surgery, University of Louisville, Louisville, KY 40202, USA; Department of Neurological Surgery, University of Louisville, Louisville, KY 40202, USA; Department of Neurology, University of Louisville Health, Louisville, KY 40202, USA; Department of Neurology, University of Louisville Health, Louisville, KY 40202, USA; Department of Neurological Surgery, University of Louisville, Louisville, KY 40202, USA; Department of Neurological Surgery, University of Louisville, Louisville, KY 40202, USA; Department of Neurological Surgery, University of Louisville, Louisville, KY 40202, USA; Department of Neurological Surgery, University of Louisville, Louisville, KY 40202, USA; Department of Neurological Surgery, University of Louisville, Louisville, KY 40202, USA; Department of Neurological Surgery, University of Louisville, Louisville, KY 40202, USA

**Keywords:** subthalamic nucleus, local field potentials, conflict control

## Abstract

The subthalamic nucleus is thought to play a crucial role in controlling impulsive actions. Networked among the basal ganglia and receiving input from several cortical areas, the subthalamic nucleus is well positioned to influence action selection when faced with competing and conflicting action outcomes. The purpose of this study was to test the dissociable roles of the dorsal and ventral aspects of the subthalamic nucleus during action conflict in patients with Parkinson’s disease undergoing intraoperative neurophysiological recording and to explore a potential mechanism for this inhibitory control. We hypothesized that modulations of neurophysiological activity during action conflict would be more pronounced in the dorsal subthalamic nucleus compared with the ventral subthalamic nucleus, due to the dissociation of cortical afferents to subthalamic nucleus subregions and previous findings of deep brain stimulation targeting subthalamic nucleus subregions in Parkinson’s disease. We recorded neurophysiological activity while 10 participants with Parkinson’s disease performed the Simon task during deep brain stimulation surgery. Response-locked local field potentials in the theta and beta band (associated with conflict control and movement inhibition, respectively) were analysed across subthalamic nucleus subregions and hemispheres relative to the motor response (ipsilateral/contralateral). In the presence of action conflict, the dorsal subthalamic nucleus, connected to cortical motor regions, exhibited larger theta power relative to the ventral subthalamic nucleus subregion, which is linked to the limbic circuits (*P* < 0.05). This evidence supports independent subregion function in conflict control. However, both subregions had relatively increased beta power for conflict trials compared with non-conflict in the hemisphere ipsilateral to the motor response. The conflict-related beta modulation was not present in the contralateral hemisphere. This indicates the importance of the ipsilateral hemisphere in the inhibition of incorrect action impulses. Additionally, higher intertrial beta power in the ventral subregion correlated with reduced accuracy on conflict trials, which we propose, could serve as a biomarker for impaired task performance. The results of the study support the existence of a functional dissociation within subthalamic nucleus subregions, emphasizing the role of the dorsal subthalamic nucleus in modulating action conflict.

## Teaser

Bowersock *et al*. reported functional dissociation in subthalamic nucleus subregions through differing theta and beta oscillations with conflict. Conflict elicited greater theta power in the dorsal subthalamic nucleus, while higher intertrial ventral beta power correlated with reduced accuracy. Both subregions showed increased ipsilateral beta power on conflict trials.

## Graphical Abstract

**Graphical Abstract:**
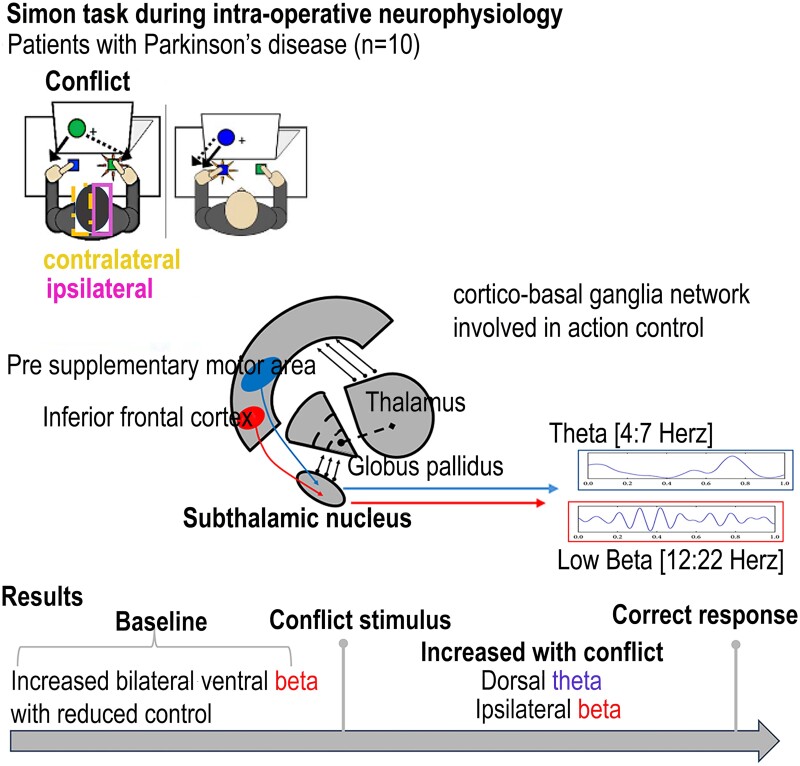

## Introduction

The cortico-basal ganglia network is an important circuitry of convergence for motor, executive and limbic processing. One theorized function of this convergence is to influence the selection and suppression of response options that are concurrently activated and competing in the motor system.^1-4^ The manner in which competing motor plans are resolved has been tied to the existence of distinct pathways traversing the cortico-basal ganglia network.^5-7^

The subthalamic nucleus (STN) is a key node in the indirect and hyper-direct pathways of the cortico-striatal network, which has led to several conceptualizations about its role in suppressing action.^8^ For example, the role of the STN in the indirect pathway is to provide a mechanism for suppressing competing actions so that a desired action can be amplified for selection by the direct pathway.^9,10^ The hyper-direct pathway, composed of motor and premotor cortical fibres directly projecting to the STN, offers a short latency signalling pathway by which STN may exert rapid inhibitory control over actions.^11^ This global suppression signalling may have a direct effect on raising the selection threshold to further deflect conflicting response options and allow only the most amplified and reinforced response option to prevail.^12,13^ Alternatively, it has been proposed that the hyper-direct pathway provides a route for rapid termination to abruptly stop all actions (global inhibition).^14,15^

Stimulation studies affirm that the STN plays a direct role in suppressing actions. Several investigations have demonstrated that deep brain stimulation (DBS) of the STN in patients with Parkinson’s disease alters their ability to abruptly stop their actions and suppress action impulses that conflict with selection of a goal-directed response.^16-18^ Specifically, it has been demonstrated that low-amplitude stimulation of the dorsal STN effectively restores conflict processing and performance to a greater extent that similar stimulation in the ventral subregion.^19^ As an established relay station within the basal ganglia, the STN receives several afferent cortical projections. Structurally, the dorsal subregion with motor dominant functions is innervated by cortical projections from the primary motor cortex (M1) and supplementary motor area (SMA).^20-22^ The relatively more ventral region mediating processes within the cognitive and limbic pathways^23,24^ receives projections from pre-SMA, inferior frontal cortex and dorsal lateral prefrontal cortex.^25,26^ However, how these dissociable networks are recruited during action control has not been demonstrated yet. The purpose of this study was to understand the relative contribution of the dorsal and ventral aspects of the STN during action conflict processing in patients with Parkinson’s disease. Conflict processing reportedly engages both motor (SMA) and cognitive/limbic cortico-striatal networks and structures (pre-SMA and anterior cingulate cortex). Likewise, the prevalence of theta and beta oscillations during action control in the STN and separately across cortical networks has been relatively well established.^2,27^ Frontal striatal theta oscillations are crucial in conflict detection by signalling the need to resolve discrepancies between competing actions or responses.^9,28^ Beta oscillations, on the other hand, are associated with movement cessation; increases in beta power inhibit ongoing motor activity.^29^ Given the dissociation of cortical afferents to STN subregions and findings from previous DBS studies on STN subregions in patients with Parkinson’s disease,^19,30,31^ we hypothesized that the dorsal, relative to ventral STN, would have a more influential role in regulating action conflict control. We further hypothesized that this would be reflected in the modulation of theta^3^ and beta^27^ power following the presentation of conflicting choices.

The STN is a recognized and effective target for the therapeutic placement of a DBS electrode in the treatment of Parkinson’s disease motor symptoms.^31,32^ As such, this population presents the opportunity to record and evaluate the role of the STN in action control. We recorded bilateral intraoperative neurophysiology within the dorsal and ventral STN in Parkinson’s disease patients undergoing DBS surgery while they performed a Simon task. We tested the hypothesis that beta and theta signalling in STN will be modulated by conflict and differ across subregions. We aim to explore the functional differences between anatomical and functional aspects of the STN in the presence of a conflicting action decision using the Simon task.^33^ This cognitive paradigm is widely acknowledged in cognitive neuroscience and measures the interference that arises between an automatic action impulse and a goal-directed action. The manifestation of interference is characterized by prolonged reaction times (RTs) and reduced accuracy (Acc).^19,31,34-36^ This study aims to further establish the role of the STN in conflict control and could contribute to a more refined understanding of DBS effects on cognition that guide future neuromodulates therapies.

## Materials and methods

### Participants

The study included 13 patients with Parkinson’s disease who received STN-DBS. Of the initial 13 patients, 11 completed the full protocol, and one was excluded based on data quality. A final sample of 10 patients (one female) was used for analysis (Table 1). Inclusion criteria involved eligibility for STN-DBS surgery as confirmed by our movement disorder multidisciplinary case conference. All patients had been deemed good candidates for STN DBS surgery based on standard clinical practice. Exclusion criteria for the research included a neurological condition other than Parkinson’s disease, psychiatric disorder including bipolar disorder or schizophrenia, or a medical condition that affects cognition (i.e. prior brain surgery and traumatic brain injury).^31^ All participants provided informed consent before participating in the study in compliance with the standards of ethical conduct in human investigation as regulated by the University of Louisville (IRB 17.0307). Participants were withdrawn from medication during the surgery as clinically required.

**Table 1:** Demographic data (mean and standard deviation) for the Parkinson’s disease patients included in the study

	Demographics (*n* = 10)
	**Mean (SD)**
Age	65.30 (6.83)
Years of education	14.33 (3.00)
Disease duration	7.10 (3.51)
UPDRS OFF	34.89 (15.99)

### Neurophysiology recording

The DBS surgery was performed with stereotactic microtargeting platform (FHC Inc., Bowdoin, ME, USA).^37^ This device provided a rigid registration of anatomical targets identified on magnetic resonance imaging (MRI) to fixed skull positions, providing precise positioning of microelectrodes and therapeutic DBS electrodes. Microelectrode recording was used to confirm STN penetration and to overcome any intraoperative shift in target position due to cerebrospinal loss or brain shift.^38-40^ Arrays of three tungsten microelectrodes (0.3–1.0 MΩ at 1 kHz; Model 44970R; FHC Inc., Bowdoin, ME, USA) were attached to each microdrive via guide mini cannulas spaced 2 mm apart in a ‘Ben-gun’ configuration.^41,42^ Six patients had bilateral recordings, a common practice at our centre, where both hemispheres were mapped simultaneously. One patient had unilateral-hemisphere recordings (left hemisphere). One patient had these procedures performed iteratively in a single surgical procedure, first the left hemisphere and then the right hemisphere. The remaining patient had staged surgeries, first receiving a left hemisphere implant followed by the second, right hemisphere, device implanted a week later. For each patient, a 10-s microelectrode recording was made at regular intervals (every 0.5 mm) along a predefined trajectory to the STN starting 10 mm above the preoperatively defined target and finishing 5–8 mm below target, usually a few millimetres inferior to the dorsal border of the substantia nigra. Recordings within the thalamus, zona incerta, STN and substantia nigra were classified by an experienced neurophysiologist. The dorsal border of the STN was classified using accepted criteria, namely, increased background activity (neuronal ‘hash’) and high-rate irregularly firing neurons.^37,43,44^ The ventral STN border was identified by the reduction of background activity and subsequently confirmed by the increase in spike amplitude indicating the substantia nigra. The ventral recordings were always conducted before we had passed the ventral border (i.e. we did not back up electrodes to perform ventral recordings). The dorsal recording depth was on average 1 mm after encountering the dorsal border of STN. The ventral recording depth was on average 3.8 mm below the dorsal entrance point of STN. See Supplementary Fig. 4 for a visualization of the recording sites in the STN.^45^ The system recorded from all channels simultaneously. Three microelectrodes per hemisphere are typically advanced along planned trajectories. For bilateral cases, up to six microelectrodes were advanced simultaneously.^46^ Microelectrode recordings were acquired along the dorsal to ventral STN axis with an Alpha Omega data acquisition system (Alpha Omega Engineering, Nazareth, Israel). This system provided recording at 44-kHz sampling for three channels per hemisphere. Accessory digital and analogue channels integrated time-locked stimulus and response inputs from the Simon task.

### Behavioural task

Participants viewed the Simon task intraoperatively on a mounted screen while lying supine. The Simon task was run through the MonkeyLogic MATLAB interface^47,48^ and the computer presenting the task was connected to the Alpha Omega to send event triggers (timestamps)—trial start, cue presentation, response and trial end—to the neurophysiology data recording system.

The first block of Simon task was administered close to the dorsal border of the STN as defined by microelectrode recording. The Simon task required a directional response to a feature (colour) of a spatially lateralized stimulus. Lateralized coloured circle stimuli are sequentially presented left or right of a central fixation point. Using handheld response grips with a button trigger in each hand, participants were instructed to make a left or right response corresponding (Cs) to the circle colour (i.e. press left to a green; press right to a blue circle arrow) as fast and as accurately as possible. The side of the stimulus presentation (relative to a central fixation point) evokes a prepotent response bias to that direction; i.e. left side presentation evokes a left-hand response. When the action signalled by the spatial location and colour of the stimulus are non-corresponding (NC), inducing conflict, the inappropriate action impulse must be suppressed. Cs trials present the stimulus on the same side as the correct response indicated by the colour rule. When a coloured circle appeared to the left or right of a central fixation point, it remained on the screen until the participant either made a response, or a time limit of 1000 ms was reached, upon which the circle was extinguished and the variable intertrial interval of 750–1250 ms elapsed before another circle appeared. If a participant made a response prior to a circle appearing, ‘TOO EARLY’ appeared in black text, and the next trial would reset. The fixation point always remained on the screen. Thus, each trial was defined by a fixation point that after a variable intertrial interval was, followed by the presentation of the imperative arrow until a response was issued or the time limit elapsed. Patients performed up to four blocks of the Simon task, with two blocks (144–190 trials per block) at each depth (dorsal and ventral STN), as tolerated, while activity was recorded from bilateral microelectrodes. Neural signals collected during intraoperative recording sessions were digitized at 44 kHz and saved for offline analysis. Digitized waveforms and stimulus events (timestamps) were saved for offline analysis. Neurophysiological analyses were performed in MATLAB (MathWorks, Natick, MA, USA) to calculate significant changes in the firing pattern in relation to stimulus conditions.

### Neurophysiological data processing

The raw signal (from the microelectrodes) was retrieved from the Alpha Omega neurophysiology recording system and analysed with custom-built MATLAB code. The overall signal was cut into trials, and correct trials were organized based on correspondence (task condition), subregion (dorsal/ventral) and hemisphere (ipsilateral/contralateral). From the three channels of data (per hemisphere), the channel with the highest variability within the trial period was selected and all three channels were used to demean the data.^49^ Data were downsampled to 1000 Hz, detrended and filtered between 1 and 100 Hz, second ordered and notch filtered between 59 and 61 to isolate the local field potentials (LFPs).^27^ Data were Fourier transformed, and wavelets with range cycles of [4 12] were applied to the analytic signal. The signal was decomposed into theta [4:7 Hz] and low beta [12:22 Hz] bands. Low beta specifically has been found to be modulated by movement in this population; thus, our analyses focused on this low beta range.^50^ Baseline values of theta and beta activity were taken from the variable intertrial period for each subregion of the STN (dorsal/ventral) and decibel normalized. This baseline activity consisted of 380 ms selected between 500 and 100 ms before the timestamped button response.

All correct trials, across conditions, within frequency bands were tested against the baseline period preceding the specific trial. To isolate condition effects, only the trials that were significantly different from the full spectrum baseline period using a one-sample paired sample *t*-test were included for further statistical analysis.^27^ The excluded trials accounted for <6.7% of all the trials across both conditions (5.76% of Cs trials and 6.65% of NC trials). The trial period was aligned to the response, *z*-scored against the band-specific baseline on a per-subject basis. An average was computed across four equal-sized time bins for each participant on the 380 ms before response to get a power score within the bands across both hemispheres (ipsilateral/contralateral). This time window was selected to avoid periods of limited to no data given the variability of responses across trials and subjects. The hemisphere (ipsilateral/contralateral) was determined relative to the response hand laterality.

### Statistical analysis

#### Behaviour

RT latencies for NC and Cs trials faster than 150 ms (anticipatory reactions) and slower than 3 SD of the mean within each condition were excluded from data analysis. Mean RTs and Acc rates were calculated for NC and Cs trials. Additional measures from distributional analyses were used to provide more insight into the dynamics of the control process across the RT distribution.^30,51,52^ We rank-ordered single-trial RTs from fastest to slowest and then divided these into four equal-sized bins.

Based on the dual process activation suppression model and our previous findings with this model,^19,30,31,52-56^ we were interested in two measures from the distributional analyses: fast response impulses (error rates) and inhibition of incorrect responses. Fast response impulses were captured by plotting Acc rates across the RTs for NC and Cs trials. The proportion of errors in the fastest RT bin on NC trials reflects the strength of impulse capture. Next, we calculated the RT interference induced by NC relative to Cs trials across the RT distribution. The reduction of interference from NC trials at the slow end of the RT, i.e. the final suppression slope, has been shown to be the most sensitive metric of inhibitory control proficiency.^30,52,53^ The full distribution plots for impulse suppression and conditional Acc are added to the supplementary document (Supplementary Fig. 1).

We used *t*-tests to compare performance on NC with Cs trial types (RT and Acc) separate for dorsal and ventral task performance. Inhibitory control measures from the distribution analyses (delta slopes and impulsive capture) were compared between dorsal and ventral recording blocks.

#### Neurophysiology

LFP values were log transformed because the analytical model assumptions were not satisfied with untransformed LFPs. For each frequency band (theta and beta), we used a generalized mixed linear model to test whether the power in the LFP band depended on the STN subregion, conflict or hemisphere. Independent variables were STN subregion (dorsal STN and ventral STN), correspondence (Cs and NC) and hemisphere (contralateral and ipsilateral) and two-way interactions between STN subregion and correspondence, between hemisphere and correspondence and a three-way interaction between STN subregion, correspondence and hemisphere. The models included a random intercept per individual, a random slope for time (four equal-sized time bins) nested within the correspondence, which itself along with hemisphere was nested within STN subregion, and a random slope for correspondence.

Estimates of each condition were presented in the log-transformed scale along with the associated standard error. Different comparisons of interest were obtained by constructing linear contrasts on the three-way interaction. All tests were two sided, and the significance level was set to 5%. The analysis was performed in SAS 9.4 (SAS institute, Cary, NC, USA).

To investigate whether individual differences in baseline beta and theta power could explain variability in action control performance, we used Spearman correlations between baseline beta and theta (separate for each STN subregion, averaged across hemispheres) and conflict control measures (Simon effect RT and per cent Acc).

## Results

### Behaviour

The Simon effect (difference between Cs and NC trials) was present in both dorsal and ventral recordings; performance was significantly slower (42.1 ms dorsal and 34.8 ms ventral) and less accurate (error rates 6.4% dorsal and 5.0% ventral) for NC compared with Cs trials in both dorsal and ventral performance blocks [*t*_RT_Do_(7) = 3.74, *P*_RT_Do_ = 0.007, Cohen’s *d* = 1.32; *t*_Acc_Do_(7) = 2.42, *P*_Acc_Do_ = 0.046, Cohen’s *d* = .86; *t*_RT_Ve_(9) = 2.30, *P*_RT_Ve_ = 0.047, Cohen’s *d* = 0.73; *t*_Acc_Ve_(9) = 2.01, *P*_Acc_Ve_ = 0.075, Cohen’s *d* = 0.64]. See Table 2 with means and standard errors for each condition.

**Table 2:** Mean and standard errors of RTs (in ms) and Acc (in % correct) of Simon task performance with dorsal and ventral recordings

	Dorsal		Ventral	
	RT	Acc	RT	Acc
Cs	583.0 (42.5.6)	88.54 (4.9)	568.2 (38.1)	89.32 (3.8)
NC	625.1 (42.2)	81.89 (6.3)	599.2 (32.4)	84.98 (4.7)
Final delta slope (NC-Cs final RT bin)	0.08 (0.05)		−0.11 (0.09)	
NC Acc first RT bin		71.07 (7.4)		69.01 (7.1)

The distribution analyses additionally indicated that impulsive errors (fast errors from the conditional Acc function on NC trials; see Supplementary Fig. 1A and B) and selective suppression (reflected by the final delta slopes; Supplementary Fig. 1C and D) were similar between subregions, *t*_slope_(7) = 2.32, *P* = 0.05, Cohen’s *d* = 0.82, *t*_AccBin1_(7) = 1.47, *P* = 0.19, Cohen’s *d* = 0.52. Note that the suppression slope during dorsal task performance was not as negative going (indicating less proficient suppression) as with ventral task performance, although this difference was not significant.

### Neurophysiology

#### Theta power modulations during conflict control

**Figure 1:**
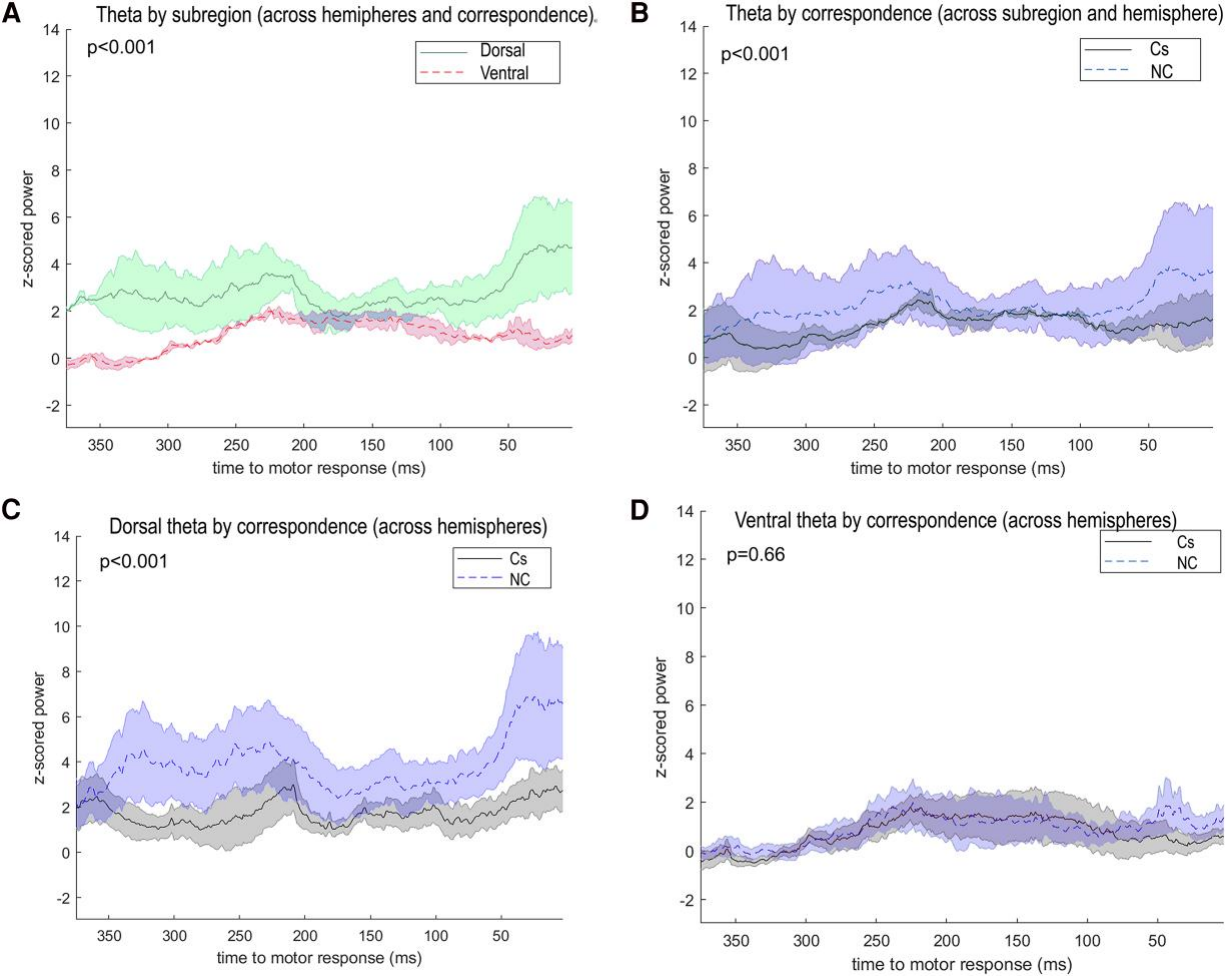
**Theta band modulations.** Normalized theta power plotted across time (ms) before the response (*n* = 10) was significantly increased in (**A**) dorsal compared with ventral STN (across correspondence and hemispheres), generalized linear mixed model (GLMM), *F*(1, 280) = 24.80, *P* < 0.001, (**B)** NC (blue dashed line) trials compared with Cs (black solid line) (across STN subregions and hemispheres), GLMM, *F*(1, 280) = 17.02, *P* < 0.001, (**C**) dorsal NC trials compared with Cs trials, GLMM-specific contrast, *t*(280) = 4.20, *P* < 0.001, **(D**) but no difference was present between NC and Cs trials in the ventral STN, GLMM-specific contrast, *t*(280) = 1.41, *P* = 0.16.

Figure 1 displays significant main and interaction effects on theta power between STN subregions and correspondence effects between STN subregions.

The mixed linear model showed that theta power was higher in dorsal compared with ventral STN [STN subregion, *F*(1, 280) = 24.80, *P* < 0.001, *m*_dorsal_ = 0.74, *m*_ventral_ = 0.64] and higher in NC relative to Cs trials [Correspondence, *F*(1, 280) = 17.02, *P* < 0.001, *m*_Cs_ = 0.65, *m*_NC_ = 0.73]; see Table 3 for the mean theta power by condition. There was no significant main effect of hemisphere [*F*(1, 280) = 0.17, *P* = 0.68] (Supplementary Fig. 2a). Moreover, there was a significant interaction between correspondence and STN subregion [*F*(2, 80) = 5.40, *P* = 0.02]. A more detailed inspection of this interaction shows that within the dorsal STN, theta was relatively more increased on NC (*m*_NC_ = 0.80) versus Cs trials (*m*_Cs_ = 0.68) (*t*(280) = 4.20, *P* < 0.001), whereas there was no correspondence effect in the ventral subregion [*t*(280) = 1.41, *P* = 0.16, *m*_NC_ = 0.66, *m*_Cs_ = 0.62] (Supplementary Fig. 2b). There were no additional two- or three-way interactions between the factors (*F*s < 1.94, *P*s > 0.16).

**Table 3:** Mean log transformed theta power (standard errors) before the response for each Simon task condition by STN subregion and hemisphere

Theta	Dorsal		Ventral	
	Ipsi	Contra	Ipsi	Contra
Cs	0.67 (0.04)	0.68 (0.03)	0.64 (0.03)	0.60 (0.04)
NC	0.76 (0.04)	0.84 (0.03)	0.66 (0.03)	0.65 (0.03)

#### Beta power modulations during conflict control

**Figure 2:**
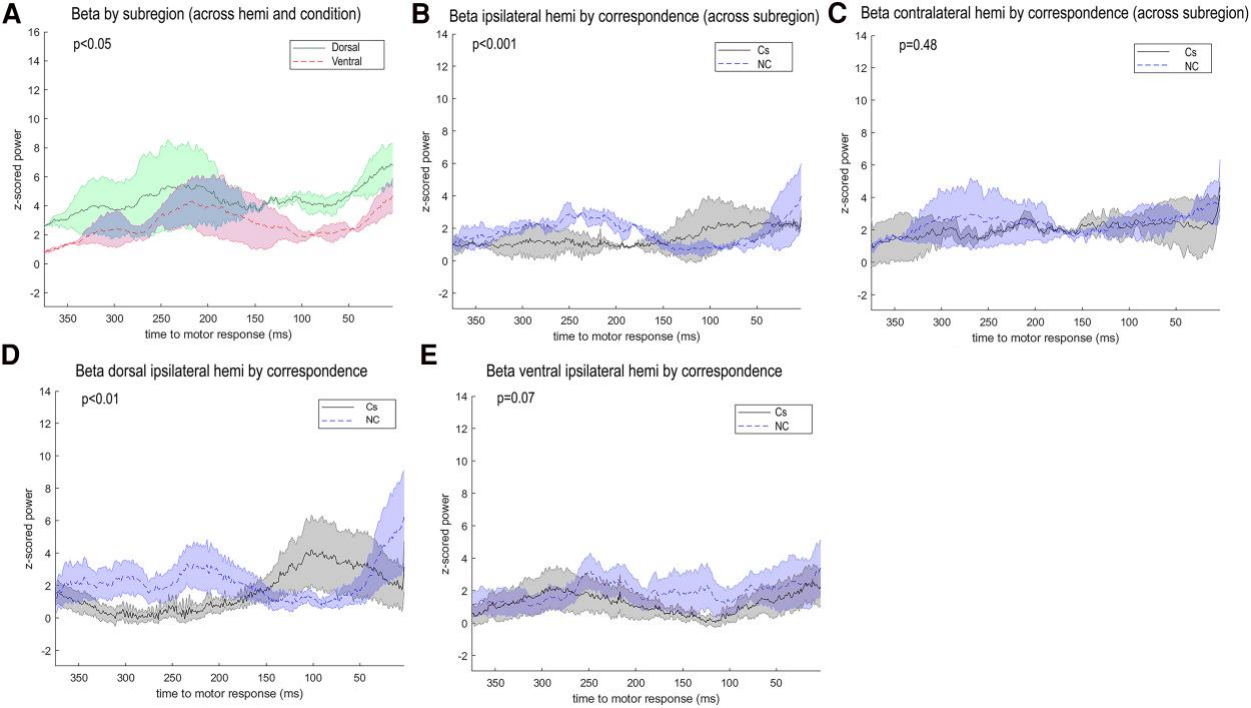
**Beta band modulations.** Normalized beta activity plotted across time (ms) before the response (*n* = 10) (**A**) was significantly enhanced in the dorsal compared with ventral STN subregion (across correspondence and hemispheres), generalized linear mixed model (GLMM), *F*(1, 280) = 4.97, *P* = 0.03, was (**B**) increased in NC (blue dashed line) versus Cs (black solid line) trials in the ipsilateral hemispheres (across subregions), GLMM-specific contrast, *t(*280) = 3.25*,P* < 0.001, (**C**) but was not different when comparing NC and Cs trials in the contralateral hemisphere, GLMM-specific contrast, (*t*(280) = 0.70, *P* = 0.48. The difference between NC and Cs trials was present in the (**D**) dorsal ipsilateral and (**E**) ventral ipsilateral hemispheres.

Figure 2A–E displays significant main and interaction effects from the mixed model analyses on comparing the beta power between STN subregions and contrasting the correspondence effect in ipsilateral and contralateral hemispheres by STN subregion.

The mixed linear model demonstrated that beta power was significantly higher in the dorsal compared with ventral STN [STN subregion, *F*(1, 280) = 4.97, *P* = 0.03, *m*_dorsal_ = 1.64, *m*_ventral_ = 1.54]. See Table 4 for the mean beta power by condition. Moreover, there was a two-way interaction between hemisphere and correspondence [*F*(1, 280) = 6.65, *P* = 0.01]. Beta was significantly lower in Cs (*m* = 1.47) relative to NC trials (*m* = 1.65) in the ipsilateral hemisphere [*t*(280) = 3.25, *P* < 0.001], but there was no difference between Cs and NC trials in the contralateral hemisphere [*t*(280) = 0.70, *P* = 0.48, *m*_Cs_ = 1.60, *m*_NC_ = 1.65]. There were no significant main effects of hemisphere and correspondence (*F*s < 2.21, *P*s > 0.14) (Supplementary Fig. 3A and B).

**Table 4:** Mean log-transformed beta power (standard errors) before the response for each Simon task condition by STN subregion and hemisphere

Beta	Dorsal		Ventral	
	Ipsi	Contra	Ipsi	Contra
Cs	1.45 (0.07)	1.75 (0.09)	1.48 (0.06)	1.55 (0.08)
NC	1.65 (0.07)	1.73 (0.07)	1.65 (0.07)	1.47 (0.08)

Additionally, there was a three-way interaction between correspondence, STN subregion and hemisphere [*F*(2, 280) = 3.58, *P* = 0.03]. In the ipsilateral hemisphere, beta power in both dorsal and ventral subregions was significantly larger on NC compared with Cs trials [*t*(280) > 2.28, *P*s < 0.05]. In contrast, in the contralateral hemisphere, beta was elevated in the dorsal relative to the ventral subregion across NC and Cs trials [contralateral hemi: dorsal NC-ventral NC, *t*(280) = 2.73, *P* < 0.01; dorsal Cs-ventral Cs, *t*(280) = 1.82, *P* = 0.07].

#### Correlations between neurophysiology and cognition

**Figure 3:**
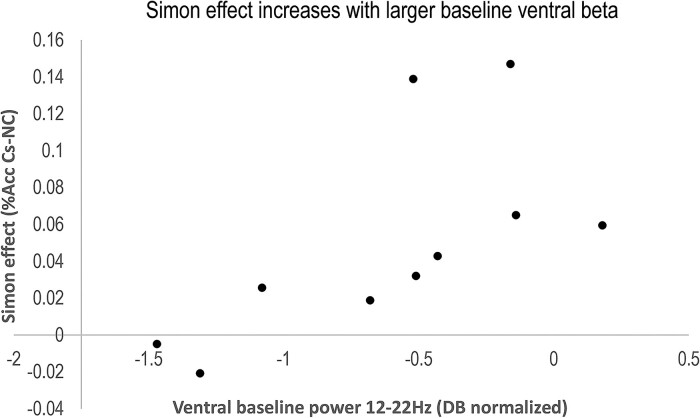
**Correlation between behaviour and baseline beta.** Ventral baseline beta power [decibel (DB) normalized, *n* = 10] positively correlates with the Simon effect (difference between Cs (black solid line) and NC (blue dashed line) trials in percentage correct), Spearman’s rho, *r* = 0.78, *P* = 0.008. Each point represents average ventral baseline beta power and Simon task performance from an individual participant. More ventral baseline beta power was associated with a larger Simon effect, indicating impaired inhibitory control with higher baseline beta.

Figure 3 shows the correlation between the ventral baseline beta power and the Simon effect in Acc during task performance with the ventral recordings.

The baseline correlations in Table 5 indicated that ventral baseline beta power significantly correlated with the Simon effect (difference in % correct on Cs and NC trials) (*r* = 0.78, *P* = 0.008). Larger baseline beta power in the ventral subregion was associated with reduced task performance as reflected by the Simon effect. None of the other correlations were significant (*r*s < 0.3, *P*s > 0.37).

**Table 5:** Correlations between baseline theta and beta power by STN subregion with action control measures

Baseline	Dorsal		Ventral	
	Theta	Beta	Theta	Beta
Simon effect RT	−0.24	0.26	0.08	0.32
Simon effect Acc	−0.26	−0.26	0.12	0.78**

**P < 0.01.

## Discussion

The STN is a crucial node in the cortico-striatal thalamo-cortical network involved in implementing control over conflicting actions.^54,55,57,58^ However, associations between the functional subregions STN and conflict control have yet to be clearly established. The current study measures intraoperative LFPs in Parkinson’s disease patients from the dorsal and ventral STN, in patients with Parkinson’s disease, during an action conflict resolution task. This improves our understanding of the function of STN subregions within the associated cortico-striatal thalamo-cortical networks.^1-4,19,30^ We hypothesized that dorsal, relative to ventral, STN would have a greater role in action control, which would be reflected by modulations of theta and beta LFP oscillation patterns.

In our study, patients with Parkinson’s disease that performed the Simon task in the OR displayed slower RTs on NC trials compared with Cs trials, successfully replicating previous findings with Parkinson’s disease patients.^19,30,31^ Importantly, theta oscillations were significantly higher in NC trials as compared with Cs trials, and this task-related modulation was most prominent in the dorsal STN. Beta power was also higher in the dorsal compared with the ventral subregion of the STN, irrespective of conflict. Modulation of beta power additionally varied between hemispheres and subregions. Within the contralateral hemisphere, there was more dorsal compared with ventral beta power regardless of trial condition (Cs or NC). However, within the ipsilateral hemisphere, there was a conflict-related increase in beta activity for NC trials in both subregions of STN. This suggests that conflicting action impulses may be regulated by beta activity in the ipsilateral hemisphere.

### Theta band activity in conflict control

Our findings are the first to demonstrate that conflict-related theta enhancements are localized to the dorsal STN subregion. Theta increase in the STN, irrespective of subregion, during conflict is a consistent finding in the literature. For example, power increases have been reported, during intraoperative STN recordings, on conflict trials using the Stroop, Eriksen Flanker and decision-making tasks.^13,59-62^ Similarly, EEG studies have reported Cs conflict-related scalp oscillations in the theta range in the dorsomedial prefrontal cortex (mPFC).^13^ Moreover, functional MRI studies have tracked conflict-related activation in dorsomedial frontal cortex, dorsal cingulate and pre-SMA,^63^ suggesting that these areas, connected with the STN via white matter tracks, use theta band coherence to communicate conflict detection and resolution.^1,3,13,52,62,64-67^ Cortical areas involved in conflict processing, like the DPFC and anterior cingulate cortex, have been found to project to the dorsal and ventral STN, respectively.^26^ Our findings show that motor conflict is specifically associated with activity in the dorsal STN, which is innervated by projections from M1, SMA and pre-SMA. Given that our task looked exclusively at motor conflict, future work might consider using other types of conflict (i.e. emotion or value-based conflict) to investigate whether this engages ventral STN subregions and the associated cortical areas.^2,3,64,68^

Action conflict control relies on the resolution between an impulsive, automatic response and a goal action.^62^ The detection and resolution of the conflict result in slower response times affording adequate time to substitute a considered action for the automatic response, where a resolution failure results in error. Accumulating evidence suggests a potential mechanism for the STN’s role in cognitive control. Upon detection of conflict, the mPFC uses theta oscillations to signal for increased cognitive control.^28,69-71^ The mPFC determines which actions are appropriate and necessary.^72-74^ Within the mPFC, the pre-SMA has been repeatedly found to process conflict between competing motor plans.^63,73,75-79^ The STN receives conflict-related information from the mPFC; concurrently, the STN adjusts the timing of executed actions in order to implement the selected action among competing choices.^27^ The STN influences the temporal dynamics to delay movement until the appropriate selection of an action has been made and a motor plan has been integrated.^1,57,66,80^ Thus, in cases of conflict or abrupt environmental change, the STN suppresses the automatic action impulse and allocates additional time for processing and selecting an updated motor plan. In this manner, the mPFC-STN network facilitates cognitive control with the mPFC by performing feedback monitoring and adjusting the STN action output.^27^ The conflict-related increase in theta in the dorsal STN may serve as a subsequent communication to update the motor plan or may represent an elevated decision threshold requiring the accumulation of more evidence before an action can be selected. Similar to the current results, the importance of the dorsal STN in motor conflict control has also been demonstrated by focused DBS modulations in STN subregions^19^ and by clinical STN-DBS targeted at the motor STN.^30^

### Beta band activity in conflict control

Our findings show a conflict-related beta modulation in both ipsilateral dorsal and ventral STN. Beta oscillations have an established role in movement cessation in healthy humans, with increased power and band synchronization being associated with movement cessation.^8,29,81-84^ The low beta range has been found to be modulated by movement in Parkinson’s disease, more so than higher beta band rhythm.^50^ Specifically, the current finding demonstrates increased beta in the ipsilateral hemisphere for conflict trials. We propose this indicates an enhanced inhibition of the incorrect contralateral action impulse to prevent interference with the appropriate ipsilateral response. Evidence of transient muscle activity (EMG) in the incorrect side, preceding the correct response, has previously been found in Parkinson’s disease patients performing the Simon task.^85,86^ Incorrect response activation would translate to activity in the ipsilateral motor cortex, and this would interfere with the correct response in the contralateral hemisphere. Increased beta in the ipsilateral STN could prevent the execution of this transient action impulse.

The increased beta in the dorsal relative to the ventral subregion across task conditions could be a reflection of the elevated beta that is associated with the excessive inhibition of movement in Parkinson’s disease.^87,88^ Our findings suggest that the beta increase may be specifically related to the dorsal motor region. The lack of task modulation could be due to a ceiling effect with increased beta oscillation limiting the detectable modulation, or it could be a function of the disease.^89,90^ However, the elevated beta in the ipsilateral hemisphere that is present for conflict trials, but not with Cs trials, suggests a compensatory, guarding function against incorrect impulsive actions. This is consistent with the literature showing the role of beta in inhibition.^8,27^ The combined finding of conflict-induced increases in theta in the dorsal STN and enhanced ipsilateral beta could imply that these oscillations coordinate the detection and inhibition of conflicting action impulses, respectively.^2^ With the sustained elevation of beta power in the contralateral hemisphere, the lack of modulation may again be explained due to a ceiling effect. Alternatively, the ipsilateral hemisphere may be responsible for implementing the inhibition of an incorrect response impulse, whereas the evidence of (contralateral) movement execution is seen downstream in the globus pallidus internus.^91^ This is supported by Choi *et al.*^91^ suggesting that the globus pallidus internus is responsible for the response execution as reflected by the movement-related beta. The timing between ipsilateral and contralateral modulation in the STN and globus pallidus internus could be an area of future study. Additionally, the relation between theta and beta oscillations and their relative timing requires further testing. For example, altering the decision threshold through theta frequency stimulation, timed to a conflict event, could subsequently impact (timing) of the inhibitory beta band activity.

### Individual differences in beta and conflict control

In the present study, an increased baseline beta in the ventral STN, collected during the intertrial period, was associated with a reduced Acc on NC versus Cs trials, exhibiting impaired conflict processing. Activity in the prestimulus period has previously been cited as an important aspect of cognitive control that biases the sensory and motor processes prior to the presentation of a stimulus that requires inhibition.^92^ The correlation of elevated beta in ventral STN with decreased inhibition may act as a pre-emptive readiness to update and execute.^93^

Previous work using a different inhibitory control paradigm, the stop task, has linked increased beta activity in the STN and inferior frontal cortex with global action cancellation.^94-96^ The inferior frontal cortex projects to the relatively ventral subregion of the STN; this leads to the speculation that the degree of baseline beta in the ventral STN is reflective of a global increase in motor inhibition. Beta in the ventral STN measured during the intertrial baseline may be an indicator or potential biomarker for the subsequent ability to modulate task-dependent beta during motor conflict.^27^

### Limitations

The data in the current study was collected from Parkinson’s disease patients with dopamine depletion, which affects activity across the cortico-striatal thalamo-cortical network.^97,98^ Thus, a direct translation regarding the role of the STN in conflict control to a healthy population cannot be assumed. However, other findings regarding STN function, for example the role of STN in action stopping, have been demonstrated by LFPs and single units in Parkinson’s disease patients^14,84^ and have also been corroborated in healthy participants using functional MRI^15,99^ and in animal studies.^100,101^ Moreover, the data collected in the current study were normalized to the baseline period on a trial-by-trial basis, correcting for the expected disease-related beta increases.^2,14,59,83,102^ The average beta activity in our study did not express the typical reduction immediately prior to the response, which may be a limitation for the interpretation of these findings. This could be explained by the previously reported delay in beta reduction for Parkinson’s disease patients.^103^ Future work will need to investigate the disease-related factors (disease duration and levodopa equivalent daily dose) that could determine the timing of the beta reduction before a motor response in Parkinson’s disease. Another limitation is that the study population consisted of one female; thus, a more balanced population would allow for a greater generalization of finding implications.

The current study made inferences about cortical circuitries involved in theta and beta based on the location of the recordings in the STN. However, cortical recordings (in pre-SMA or inferior frontal cortex) in parallel with STN would be necessary for a more advanced understanding of the temporal dynamics of the cortical striatal network in conflict control.

Due to the nature of the surgery, the data collection always started in the dorsal STN and then proceeded to the ventral STN, which may additionally have biased the data. However, note that the performance on the Cs trials was similar across dorsal and ventral blocks (no significant differences between the RTs and Accs, *t*s < 1.28, *P*s > 0.12). This suggests that there were no order effects. Despite dorsal recordings always preceding the ventral data collection, the behavioural performance on the Simon task was similar and suggests that order effects, from progressive penetration, were minimal. Furthermore, although most recordings were bilateral, some patients received staged placement of the bilateral electrodes, which altered the number of times they performed the task and could have impacted the data. Finally, our sample population was limited due to the difficulties in recruiting as well as performing the task intraoperatively. Patients also had slower response times making the number of error trials negligible (as low as 1%); thus, only correct trials were analysed. Future studies should aim to investigate performance on error trials.

## Conclusion

The STN has been established as a relay station in the basal ganglia, receiving input from both indirect and hyper-direct pathways involved putatively in inhibitory action control. As such, oscillations collected from the STN provide valuable insight into mechanisms dictating action control. The pausing or action cancellation function of the STN may be implemented through dissociable cortical-STN circuits reflected in the task-related modulations seen in both theta and beta frequencies. Here, we established that an increase in theta oscillations, previously linked to conflict processing, was localized to the dorsal STN on conflict trials compared with non-conflict trials. This adds further support to the hypothesis that the dorsal STN is particularly involved in resolving conflict and interference created by fast action impulses.^3^ Moreover, we detected higher beta oscillations in the ipsilateral hemisphere on conflict trials compared with non-conflict trials irrespective of STN subregion. Given links between beta signalling and inhibitory control of movement, this further supports that STN also plays a role in the suppression of an incorrect motor impulse. Whereas the conflict-related beta increases in the ipsilateral hemisphere may facilitate resolving motor impulses, the baseline beta (in ventral STN) seems to hinder subsequent task performance. The ventral baseline beta may be a potential biomarker for impaired conflict control. An improved understanding of the STN functionality across subregions, and its associated circuitry, will perhaps help tailor individually optimized DBS settings to maximize both motor and cognitive control.

## Supplementary Material

fcaf021_Supplementary_Data

## Data Availability

Anonymized data will be available upon reasonable request by a qualified investigator. Code suite is available at https://github.com/UofL-HCN-Lab/STN-DBS.git.
