# Supplementary material for: Theta and beta power in the subthalamic nucleus responds to conflict across subregions and hemispheres
Source: Brain Commun. 2025 Jan 16;7(1):fcaf021. doi: 10.1093/braincomms/fcaf021 (PMC11775628; doi:10.1093/braincomms/fcaf021)
Supplement: fcaf021_Supplementary_Data [file fcaf021_supplementary_data.pdf]

Supplementary Materials

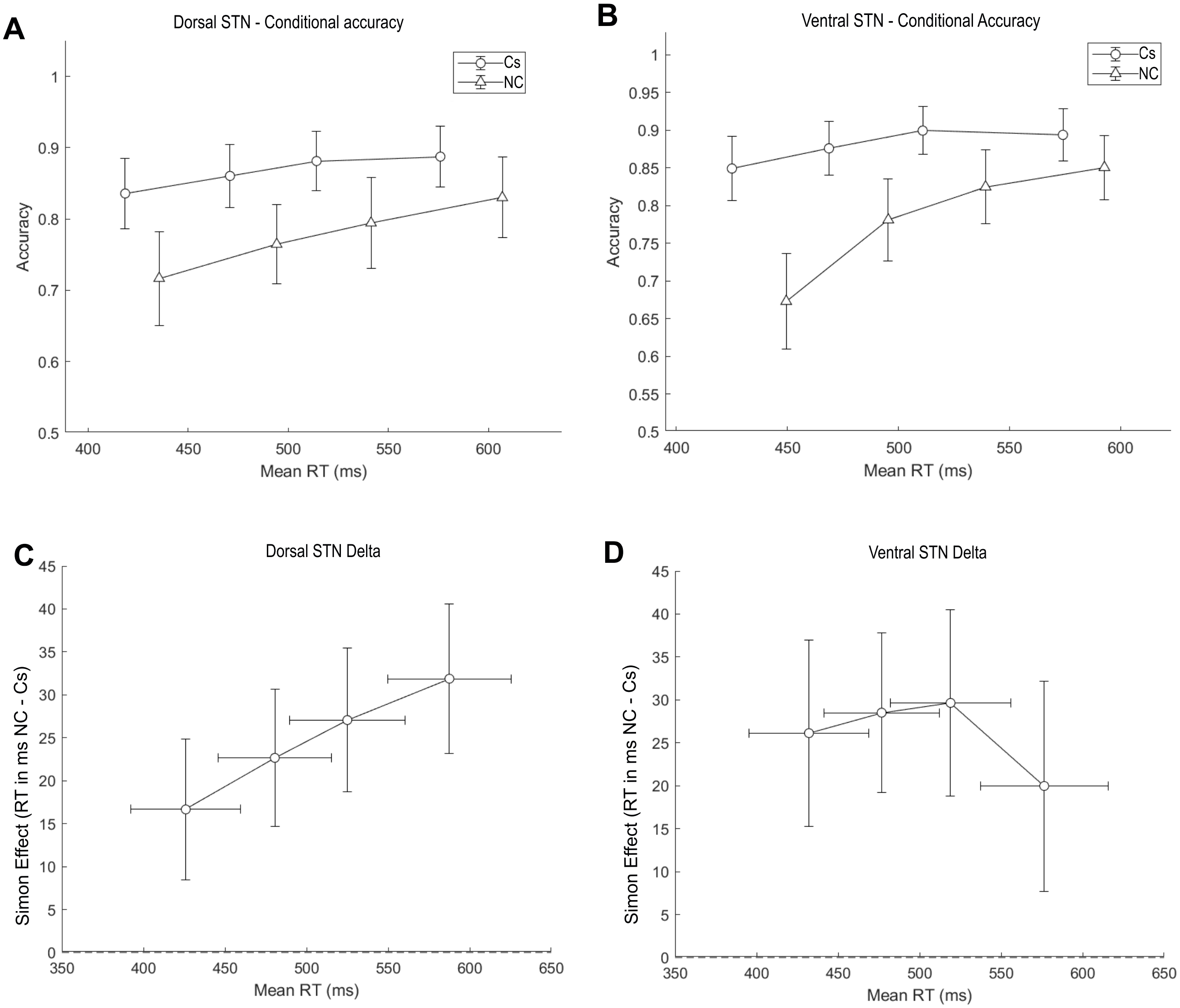

Supplementary Fig. 1. Mean and standard errors (n=10) for the conditional accuracy (CAF, probability correct) plotted across mean reaction time (RT) bins (milliseconds, ms) for corresponding (Cs) and noncorresponding (NC) trials in **a)** dorsal and **b)** ventral Subthalamic Nucleus (STN) recordings. Error rates are increased with the fastest reaction times on NC trials and were similar between subregions, paired sampled t-test, $t_{\mathrm{AccBin1}}(7)=1.47$, *P=0.19* Delta plots depict the average Simon effect (difference in ms on noncorresponding minus corresponding trials) plotted across mean reaction time bins **c)** dorsal and **d)** ventral STN recordings. Each reaction time bin contains the same number of trials, averaged across the subjects for each recording location. Note that although the delta slopes are visually different between dorsal and ventral task performance, this was not a significant difference (paired sampled t-test, $t_{slope}(7)=2.32$, $P=0.05$).

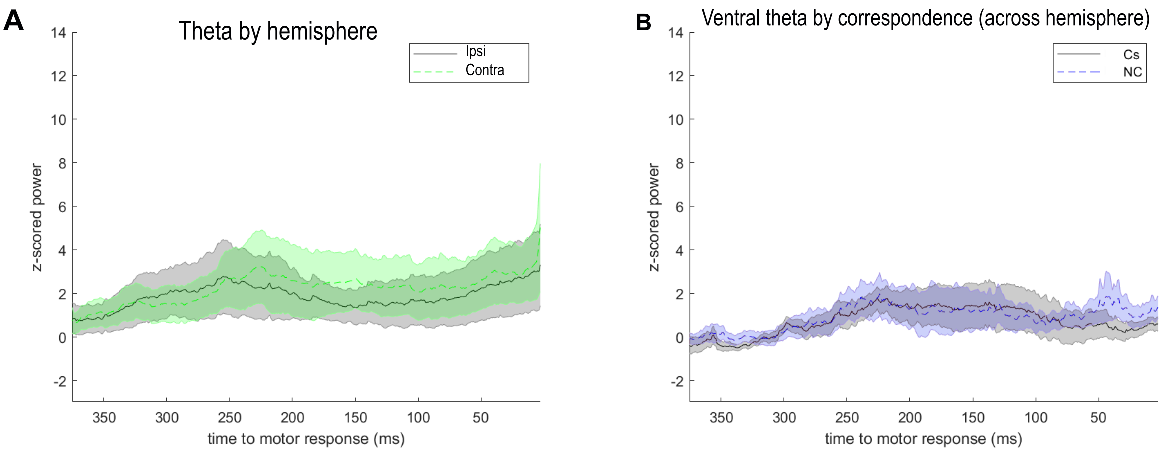

Supplementary Fig. 2. Mean normalized theta power plotted across time (ms) before the response (n=10) showed no difference between **a)** ipsilateral compared to contralateral hemisphere (across correspondence and STN subregions) generalized linear mixed model (GLMM) $F_{hemisphere}(1, 280)=0.17$, $P=0.68$, and between **b)** the ventral STN subregion (across hemispheres), comparing corresponding (Cs, black solid line) versus noncorresponding (NC, blue dashed line) trials, GLMM specific contrast, $t(280)=1.41$, $P=0.16$. Shaded areas around the mean depict standard errors.

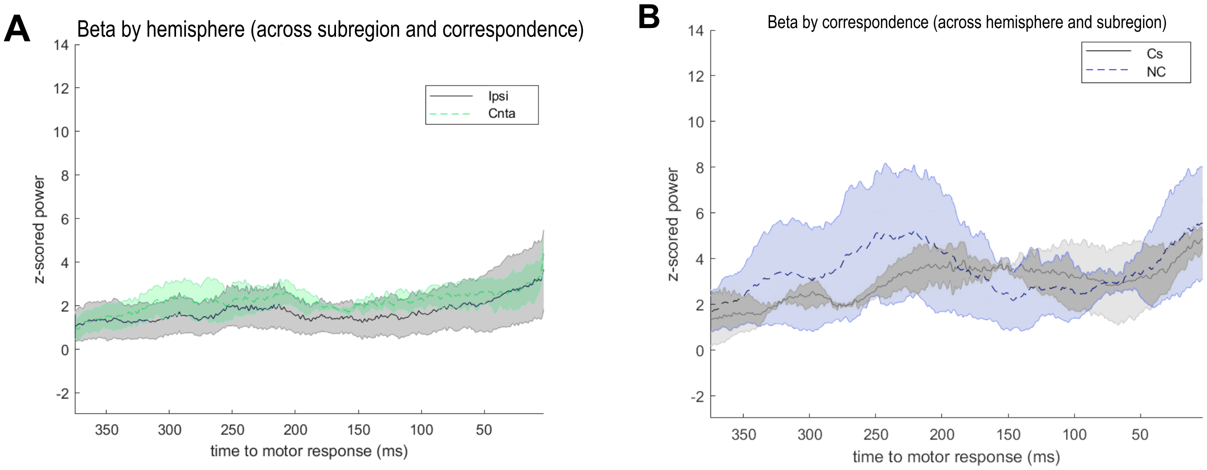

Supplementary Fig. 3. Mean normalized beta activity plotted across time (ms) before the response (n=10) showed no difference between **a)** ipsilateral compared to contralateral (across conflict and STN subregions), **b)** Corresponding (Cs) compared to noncorresponding (NC) trials (across STN subregion and hemisphere), generalized linear mixed model (GLMM) Fs<2.21, ps>0.14. Shaded areas around the mean depict standard errors.

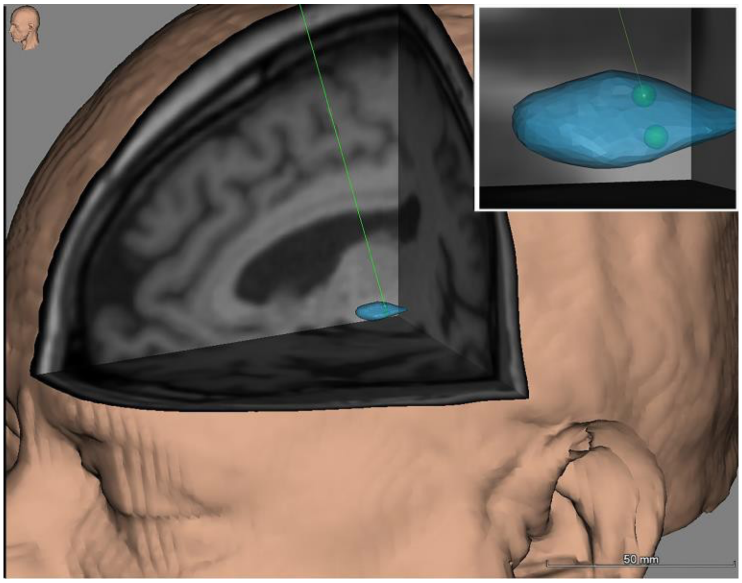

Supplementary Fig. 4. Visualization of the dorsal and ventral STN recording sites in anterior commissure (AC) - posterior commissure (PC) (AC-PC) coordinates (coordinates relative to the midcommsiural points) of a representative patient relative to the segmented atlas of the STN [1]. The enlarged insert in the top right corner (inside the white rectangle) shows the dorsal and ventral recording sites in detail. Note that the scale in the bottom applies to the overall brain, not to the enlarged STN. Mean recording locations in AC-PC coordinates for dorsal (x,y,z) was (11.4, -3.2, -3.1) and ventral (10.5, -4.4, -5.5).

References:

1. D'Haese P-F, Pallavaram S, Li R, et al. CranialVault and its CRAVE tools: a clinical computer assistance system for deep brain stimulation (DBS) therapy. Medical image analysis. 2012;16(3):744-753.
